# Acoustic Stimuli Can Improve and Impair Somatosensory Perception

**DOI:** 10.3389/fnins.2022.930932

**Published:** 2022-06-23

**Authors:** Matthias Nuernberger, Denise Schaller, Carsten Klingner, Otto Witte, Stefan Brodoehl

**Affiliations:** ^1^Department of Neurology, Jena University Hospital, Jena, Germany; ^2^Biomagnetic Center, Jena University Hospital, Jena, Germany

**Keywords:** somatosensory perception, MDT, fMRI, acoustic noise, connectivity, crossmodal interaction, white noise, sensory integration

## Abstract

The integration of stimuli from different sensory modalities forms the basis for human perception. While the relevant impact of visual stimuli on the perception of other sensory modalities is recognized, much less is known about the impact of auditory stimuli on general sensory processing. This study aims to investigate the effect of acoustic stimuli on the processing of somatosensory stimuli using real noise (i.e., unpleasant everyday noise, RN) and neutral white noise (*WN*). To this purpose, we studied 20 healthy human subjects between 20 and 29 years of age (mean: 24, SD: ±1.9 years sex ratio 1:1). Somatosensory perception was evaluated using mechanical detection threshold (MDT) of the skin on the back of the dominant hand. To investigate the underlying mechanisms in the brain, fMRI was performed while applying acoustic stimulation (RN and WN) and tactile stimulation of the dominant hand. Here we show that acoustic stimulation with noise alters the perception of touch on the skin. We found that the effect of RN and *WN* differed. *RN* leads to an improved tactile perception, whereas *WN* impaired tactile perception. These changes go along with significant differences in brain activity and connectivity. *WN* is associated with a significant increase in brain activity in multiple brain areas such as the auditory and somatosensory cortex, parietal association cortex, and the thalamus compared to *RN*. With tactile stimulation of the skin, the flow of information in these brain areas is altered. While with *RN* the information flow from the thalamus to the somatosensory cortex is prominent, the network activity pattern changes under *WN* revealing an increase in interaction between multiple networks. Unpleasant noise inhibits the multisensory integration and enables a more efficient unimodal perception in the somatosensory system, improving perception. Whether this is to be interpreted as a temporary increase in phasic alertness or by a stronger filter function of the thalamus with a preference for unimodal stimuli is still open for debate.

## Introduction

The concept of crossmodal interactions extends the classical doctrine of unimodal processing in primary sensory brain areas (Macaluso and Driver, 2005; Stein, 2012). Well-known examples of multisensory perceptual illusions caused by crossmodal interactions include the McGurk effect (McGurk and MacDonald, 1976) and the ventriloquist effect (Vroomen et al., 2001). These illusions have in common that spatial distance of otherwise associated visual and auditory stimuli lead to mislocalization and misinterpretation of sensory input. In case of contradictory information, the visual stimulus is often evaluated as more valid or a compromise between the sensory information is generated to bridge the prediction error. For example, if the spatial source of what is heard and what is seen do not match, the spatial information of what is seen can often overwrite that of what is heard. This tendency is discussed as visual dominance (Colavita, 1974). However, in addition to visual information, auditory stimuli can also influence the perception of another sensory modality (Ma et al., 2009; Kayser and Shams, 2015). The perception of high-frequency sounds leads to the perception of a surface as smoother and drier in tactile perception. Low-frequency sounds lead to a rougher and moister perception of the same surface. This effect is called parchment skin illusion (Jousmäki and Hari, 1998). Acoustic stimulation can also distract attention and impair motor learning processes (Barutchu et al., 2010). Besides specific acoustic stimuli (i.e., sounds like a car horn) and noise (i.e., a random cluster of familiar and unfamiliar sounds with pleasant and unpleasant features), the most relevant auditory research instrument is white noise (*WN*). *WN* can be described as a hissing sound likely to/h/in constant aspiration. It carries an audio signal in form of a flat spectrum across all audible frequencies. Its relevance for cognitive information processing is subject to debate, but there are also indications of beneficial aspects to learning processes (Rausch et al., 2014). Somatosensory perception can also be influenced by other sensory modalities like visual input or deprivation, as we were able to show in previous work (Brodoehl et al., 2015a,b). Closing the eyes leads to an enhanced perception of subtle touch at the expense of spatial integration of this information. The brain switches between a mode with enhanced thalamo-somatosensory coupling and a mode with enhanced multimodal integration. By extending our findings to the auditory and somatosensory systems’ interactions, we hypothesize that modulation of activity in the auditory system alters the perception of somatosensory stimuli. We assume that in addition to top-down modulation by complex stimuli, bottom-up modulation *via* crossmodal interactions also plays a relevant role. We aimed to investigate how the perception of a simple tactile touch on the skin is altered by different acoustic stimulation. The acoustic stimuli we used were a sound generally perceived as unpleasant real noise (*RN*) and a sound perceived as *WN*. To study the underlying neural mechanisms, we used functional magnetic resonance imaging to evaluate brain network activity and quantify causal information flow.

## Materials and Methods

### Participants

We recruited 20 healthy human volunteers (range: 20–29 years, mean: 23.95, SD: ±1.91 years, 10 male) without neurological or otological afflictions. All participants identified as either male or female. All participants were right-handed. Typical exclusion criteria were considered (Brodoehl et al., 2016). All subjects were informed about the procedure of the trial in written form as well as personally and gave their written consent according to the Declaration of Helsinki. All 20 recruited participants participated in all experiments described in this analysis. The trial was approved by the Ethics Committee of the Medical Faculty of the Friedrich Schiller University Jena, Germany (registration number: 4301-01/15). We performed pilot trials with five subjects to evaluate our experimental design. Data of pilot trials is not part of this analysis. The subjects of the pilot trials were matched to the planned participants of our experiments.

### Acoustic Stimuli

We used two different sounds for acoustic stimulation: *RN* and *WN*. *RN* was intended to represent very unpleasant acoustic information while *WN* was assessed as neutral to slightly unpleasant. From a variety of sounds presumed as unpleasant noise (e.g., the sound of a jackhammer, traffic noise, or people yelling), a sound sample with intense instrumental heavy metal music was selected as *RN*. It offers a wide range of frequencies and extensive temporal modulations. In a rating of 0 (not unpleasant) to 10 (very unpleasant), this sound sample was rated highest (mean: 8.3) in our pilot trials, so it was used in the final study. In the same manner *WN* was rated slightly unpleasant in our pilot trial (mean: 2.8). *WN* can best be described as a static monotonous sound without specific characteristics. Using the software program mp3Gain (developed by Glen Sawyer, Version 1.3.4), the samples was normalized to 90 dB (0.633 Pa) and a length of 10 min. As *WN*, a freely available sample which is composed of all frequencies in the human hearing range was used. It was normalized to 90 dB (0.633 Pa) and a length of 10 min as well. The volume of the acoustic stimuli was adjusted to 75 dB before application so that it was rated as loud but still tolerable by the subjects. Sound pain threshold was not reached or surpassed. As baseline apart from the forementioned two acoustic stimuli we used silence without any auditory input (*rest*).

### Somatosensory Perception Testing by Mechanical Detection Threshold

Mechanical Detection Threshold (MDT) was measured using a quantitative sensory testing (QST)-compliant set of Von-Frey-Hairs^®^ (Optihair2, Marstock Nervtest, Germany). These plastic filaments apply pressure between 0.125 and 64 mN (grating-factor: 2) when they touch the skin of the dominant backhand on a hairless spot with a diameter of 0.5 mm for 1 s while being bended to S-shape. The described procedure is standardized in our lab (Brodoehl et al., 2013). All subjects were blindfolded and received the stimulation on the same area on the back of the right hand (diameter: 1 cm, shaved area marked by colored pencil). Threshold determinations (10 stimuli each) were acquired by alternately descending until the subject failed to notice the stimulus and ascending until re-noticing occurred (“method of limits”). Means and standard errors of the results of all blocks were calculated and analyzed as surrogates for the actual threshold.

### Somatosensory Perception Experiment

**FIGURE 1:**
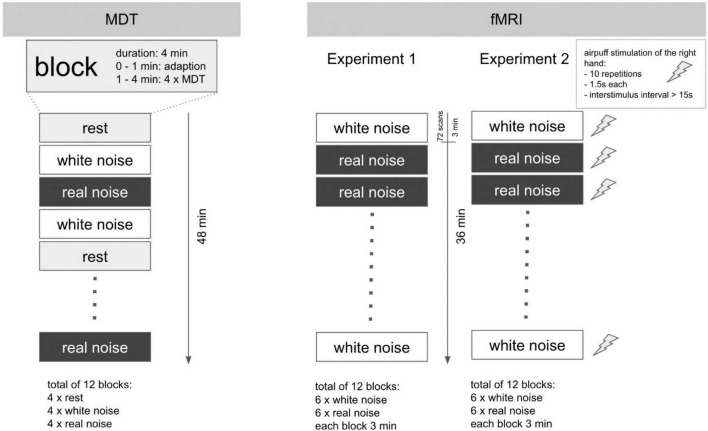
Experimental design for MDT and fMRI experiments. Schematic illustration of the experimental design. Left: MDT: a total of 12 blocks with a duration of 4 min each. The determination of the MDT was performed in each block from the second minute onward (4× per block). In sum, the study lasted 48 min per subject. Right: fMRI: the experimental setup for Experiments 1 and 2 was in major parts identical. Each of the 12 blocks with acoustic stimulus lasted 3 min. The overall duration per experiment was 36 min. In Experiment 2, however, the fingers of the right hand were additionally stimulated tactilely (*air-puff*) within each block: 10 repetitions, duration 1.5 s, interstimulus interval 15–30 s. In contrast to the MDT determination, no block with *rest* condition (silence) was performed in the fMRI experiments.

The examination took place in a darkened, anechoic room. The subjects were instructed to keep their eyes closed during the examination. Furthermore, they received a blindfold and standard noise protection headphones to prevent them from being influenced by any ambient noise. The acoustic stimuli *RN* and *WN* were presented using standard in-ear headphones. To measure the perception of a simple touch on the skin, we determined the tactile mechanical detection threshold (MDT). The actual examination was performed in a total of 12 blocks of 4 min each. During each block, either *RN*, *WN*, or no sound (*rest*) was played. The order of the blocks was arranged pseudorandomized. Within each block and starting at minute 2, MDT was determined five times. For this purpose – starting from 16 mN – the filaments were presented in descending order of strength until the subject no longer perceived any touch and then again in ascending order of strength until touch was again perceptible. The experiment is devised as a repeated measures design. A schematic representation of the procedure is shown in Figure 1 (left).

### fMRI Experiments

#### fMRI Data Acquisition Parameters

All experiments were performed using a 3.0-T MR scanner (Trio, Siemens, Erlangen, Germany) to obtain echo-planar T2*-weighted image volumes (EPI) and transaxial T1-weighted structural images. The high-resolution T1-weighted structural images had a voxel size of 1 mm × 1 mm × 1 mm to enable precise anatomical localization. EPI images were acquired using the following parameters: voxel size = 3 mm × 3 mm × 3 mm, TR = 2.52 s, TE = 35 ms, and 40 transaxial slices (including the entire cerebrum and cerebellum).

#### Experimental Setup

The fMRI examination was performed the day after the assessment of MDT. Subjects were positioned in the scanner, given standard headphones with acoustic shielding, and instructed to keep their eyes closed during the examination. After each scan, the subjects’ level of vigilance was inquired.

#### fMRI Experiment 1: Acoustic Stimulation

Experiment 1 lasted a total of 36 min. In a block design, either *RN* or *WN* was played through the headphones. The exposure to this noise in combination with the headphones makes the scanner hardly audible for the subjects. A total of 12 blocks, each lasting 3 min, were executed in a pseudorandomized order; each block consequently lasted approximately 72 EPI images. The task was passive, so no active participation of the subjects was necessary. The experiment is devised as a repeated measures design.

#### fMRI Experiment 2: Acoustic Stimulation Combined With Tactile Stimulation of the Right Hand

Duration and block design were identical to Experiment 1. Additionally, tactile stimulation with a balloon-controlled air-driven device (*air-puff*) was applied to the fingers of the right hand during the blocks. Von Frey filaments could not be used in the scanner room, because additional personal was not allowed in the scanner room during image acquisition. During 1 block, 10 stimulations occurred (interstimulus interval 15–30 s, duration 1.5 s, onset 10th second after block start). The task was passive, so no active participation of the subjects was necessary. Figure 1 (right) shows the sequence of the fMRI experiments. The experiment is devised as a repeated measures design.

#### fMRI Data Preprocessing

For each subject, all images were realigned to the first volume using six-parameter rigid-body transformations to correct for motion artifacts. The images were co-registered with the corresponding anatomical (T1-weighted) images of the subject, re-sliced to correct for acquisition delays (referenced to the 10th slice only in the event-related design), normalized to the Montreal Neurological Institute (MNI) standard brain to report MNI coordinates and smoothed using a 6-mm full-width-at-half-maximum Gaussian kernel.

### Statistical Analysis

Statistical analysis was performed using IBM SPSS27^®^ (Version: 27.0.0.0). Data was tested for normal distribution by Shapiro–Wilk and Kolmogorov–Smirnov test. Data was tested for homogeneity of variance by Levene’s test. Data was tested for equal sphericity by Mauchly’s test. Where normal distribution was attained, we used independent samples *t*-test. Where normal distribution was not attained, the Mann–Whitney *U* test was used. Where equal sphericity was not attained, Greenhouse–Geisser correction was applied. Findings were considered significant at *p* < 0.05 (two-sided). Results were corrected for family wise error (FWE) induced by multiple comparisons using Bonferroni-correction (*p* < 0.05). Standard confidence interval (95% CI: mean ± 2 SD) was used. For the evaluation of the mechanical detection threshold (MDT) in the somatosensory perception experiment we followed standardized protocol (Rolke et al., 2006): the geometric mean was calculated for each block of each stimulus (*rest*, *RN*, *WN*), and subsequently the mean was calculated for each of the stimuli. To perform repeated measures analysis of variance (repeated-measures ANOVA) logarithmic transformation of the data was performed using the natural logarithm. Data analysis for the fMRI experiments was performed on a PC using MATLAB (Version 2019a, MathWorks, Natick, MA, United States) and SPM12 software (Wellcome Department of Cognitive Neurology, London, United Kingdom^1^) (Friston, 2007). fMRI Experiment 1: multiple regression analysis using a general linear model was performed to obtain statistical parametric maps calculated for the somatosensory stimulation. The fMRI signal time courses were high-pass filtered (128 s) and modeled as an experimental-stimulus onset function convolved by the canonical hemodynamic response function (low-pass filter). Two contrasts of interest were examined, resulting in two *t*-statistical (paired *t*-test) maps (*RN* > *WN*) for the first fMRI experiment. Individual results were projected onto their respective co-registered high-resolution T1-weighted 3-D data set. The anatomical localization of the activated areas was analyzed regarding the standard stereotaxic atlas and was mapped using the anatomical toolbox of SPM12 (Eickhoff et al., 2005). Threshold free cluster enhancement (TFCE) was applied, and results were corrected for FWE induced by multiple comparisons using Bonferroni-correction (*p* < 0.05). fMRI Experiment 2: several regions of interest (ROIs) were defined based on our hypotheses. The analyzed ROIs are shown in Supplementary Table 1. The time-series data from these identified regions were extracted, and cluster-specific time series were then estimated by averaging the time series of all voxels within a cluster. Several sources of variance were removed from the data using linear regression: (1) six parameters obtained by rigid body correction of head motion, (2) a signal from a ventricular region of interest, and (3) a signal from a region centered in the white matter. All signal intensity time courses were band-pass filtered (0.01 < *f* < 0.1 Hz) to reduce the effects of low-frequency drift and high-frequency noise. Conditional Granger causality analysis (GCA) was applied to explore the dynamic causal relationship between the time series. This approach has been widely used in previous fMRI studies. In our study, GCA was performed using the toolbox implemented by Seth (2010). The detailed theory behind Granger causality has been previously described (Granger, 1969). TFCE was applied, and results were corrected for multiple comparisons using Bonferroni-correction (*p* < 0.05).

## Results

### Effects of Acoustic Stimulation Upon the Somatosensory Perception of Touch

In 20 healthy subjects (24 ± 1.9 years, 10 females) the mechanical detection threshold (MDT) was assessed under different acoustic stimuli (*rest*, *WN*, *RN*). Starting from a baseline at *rest*, 75% of the subjects showed a deterioration in perceptual performance in *WN* and 90% of the subjects showed an improvement in *RN*. In direct comparison of the two noise variants, the participants demonstrated a statistically significantly better perceptual performance with *RN* compared to *WN*, *U* = 284.000, *p* = 0.024. The mean values of the group analysis were 1.40 (±0.83 mN) for *rest*, 1.74 (±1.18 mN) for *WN*, and 1.13 (±0.55 mN) for *RN* condition. In group analysis (repeated-measures ANOVA), all pairwise comparisons were statistically significant at a significance level of *p* < 0.05 (*rest* vs. *WN*: *p* = 0.049, *rest* vs. *RN*: *p* = 0.027, and *WN* vs. *RN*: *p* < 0.001). There was no statistically significant influence age or gender. To control for fluctuations of alertness during the experiment, we tested the second block of every condition against the last block of the same condition. There was no statistically significant difference between these blocks, *p* > 0.05. Intraindividual results are shown in Table 1.

**TABLE 1:** Results of somatosensory perception testing with the mechanical detection threshold (MDT).

Subjects		Rest		WN		RN	

#	Age/sex	mN	SD	mN	SD	mN	SD
1	21/f	1.04	±0.09	1.03	±0.07	0.75	±0.05
2	22/f	1.05	±0.33	1.05	±0.31	0.78	±0.19
3	22/f	1.43	±0.23	1.98	±0.4	1.22	±0.21
4	22/f	0.71	±0.12	0.72	±0.17	0.6	±0.08
5	22/m	3.12	±0.53	2.93	±0.12	2.6	±0.27
6	22/m	1.49	±0.97	2.44	±0.93	1.23	±0.47
7	23/m	1.14	±0.73	1.75	±0.66	1.18	±0.94
8	24/f	1.8	±1.12	1.35	±0.35	0.9	±0.17
9	24/f	0.29	±0.07	0.41	±0.15	0.27	±0.08
10	24/f	1.37	±0.12	1.38	±0.41	1.35	±0.32
11	24/m	1.53	±0.26	2	±0.59	1.28	±0.09
12	24/m	1.59	±0.92	1.25	±0.08	0.83	±0.12
13	24/m	2.15	±1.35	2.54	±1.19	1.37	±1.02
14	25/f	1.02	±0.49	1.56	±0.81	0.58	±0.14
15	25/f	0.41	±0.27	0.75	±0.45	0.33	±0.18
16	25/f	0.58	±0.13	0.67	±0.27	0.33	±0.14
17	25/m	1.77	±0.65	2.26	±1.14	1.04	±0.15
18	25/m	0.64	±0.17	0.66	±0.19	0.39	±0.09
19	27/m	3.61	±0.83	5.7	±3.56	4.69	±2.35
20	29/m	1.33	±1.04	2.28	±1.85	0.91	±0.84
Mean	24	1.40	±0.83	1.74	±1.18	1.13	±0.55

*The mean value of the mechanical detection threshold (MDT) in mN is shown for each subject and each block (rest, white noise, real noise). WN, white noise; RN, real noise; SD, standard deviation.*

### Changes in Brain Activity Caused by Sound Stimulation

#### Experiment 1

**FIGURE 2:**
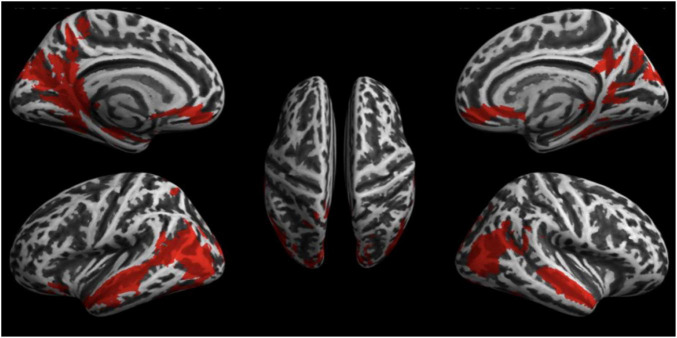
Results of the fMRI Experiment 1: WN > RN.

fMRI scans of all 20 subjects were analyzed to investigate the effect of the 2 conditions *WN* and *RN* upon brain activity (Experiment 1). The results of the second-level analysis with SPM are shown in Figure 2. Statistically significant activation (significance level at *p* < 0.05, FWE-corrected) was found only for the contrast of *WN* > *RN*. Acoustic stimulation with *WN* resulted in significantly increased brain activity patterns, especially in the temporal, parietal, and occipital cortex. Discrete activations were also detectable in the primary somatosensory cortex Clusters with increased contrast are listed in Table 2. Furthermore, increased brain activity compared to the *RN* condition was found in the hippocampus, parahippocampal gyrus, cingulate gyrus, and primary motor cortex. In summary, an increase in brain activity during *WN* was found especially in the auditory cortex and in the parietal association cortex (see Figure 2).

**TABLE 2:** Results of the fMRI Experiment 1: WN > RN.

Cluster	Voxel	*t*_*max*_	MNI			Brain regions

#	*n*		*X*	*Y*	*Z*	
1	273	7.47	−60	−26	−5	Temporal lobe left Amygdala (LB), area tE 3, area Id1 (insula), entorhinal cortex
2	161	5.7	39	4	−29	Temporal lobe right Area tE 3; area Id1 (insula)
3	117	6.08	−48	−68	19	Temporal lobe left Area PGp (IPL); area Pga (IPL), area PFm (IPL)
4	53	4.73	51	−68	19	Temporal lobe right Area PGp (IPL); area PGa (IPL)
5	52	4.44	−6	25	−20	Gyrus rectus bilateral n.d.
6	50	5.17	−15	−44	4	Gyrus fusiformis left, “lingual gyrus” Subiculum; DG (hippocampus); CA1 (hippocampus); temporal thalamus
7	38	5.07	−9	−50	52	Precuneus left Area 5m (SPL), area 5Ci (SPL), area 51 (SPL)
8	38	4.95	−12	7	−14	Olfactory cortex left, insular lobe left; IFG (p. orbitalis) left Amygdala left
9	35	4.73	6	16	−14	Gyrus rectus right, olfactory cortex right n.d.
10	32	6.47	−33	−20	−11	Hippocampus left, parahippocampal gyrus left CA3, CA2, CA1, DG (hippocampus), subiculum
11	21	4.67	−48	−80	1	Occipital lobe (inferior and medius) hOc5 (V5/Mt); area FG1; area FG2; hOc4v [V4(v)]
12	15	4.64	27	−41	4	Right hippocampus, right parahippocampal gyrus DG, CA1, CA2, CA3 (hippocampus); temporal thalamus, subiculum
13	14	4.09	12	−44	55	MCC (right cingulate gyrus) Area 5Ci (SPL); area 5m (SPL), area 3a, area 4p
14	12	4.38	6	−92	28	Cuneus right hOc3d (V3d) bds, hOc2 right (V2), hOc4d (V3A)
15	11	3.89	45	−83	10	Occipital and temporal lobes hOc5 (V5/Mt)
16	11	4.36	30	−20	−14	Hippocampus right, parahippocampal gyrus right CA1, CA2, CA3, DG (hippocampus), subiculum

*Activations are shown on an inflated brain model. Results were corrected after TFCE at p < 0.05 (FWE-corrected) and are shown in red. The contrast displayed is the pairwise t-test for the comparison: “white noise > real noise.” The table shows the number of clusters found with size (in voxels), maximum t-value, MNI coordinates, and anatomical location/description.*

#### Experiment 2

**FIGURE 3:**
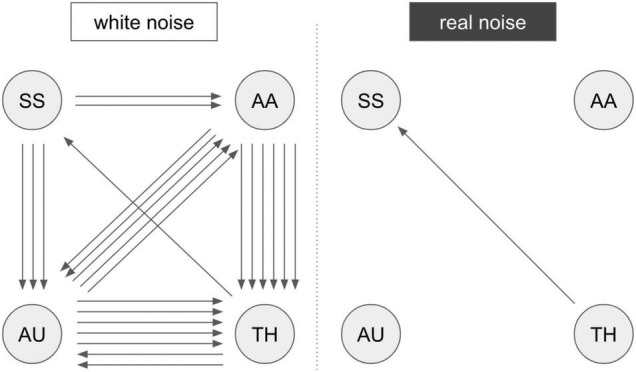
Results of the GCA analysis. Summarized results of the Granger causality analysis of fMRI Experiment 2 with pairwise comparison “white noise > real noise” (white noise) and “real noise > white noise” (real noise). The arrows represent significant connections in the comparison matrix (Supplementary Figure 1). An arrow represents a registered causal link (compare Supplementary Figure 1). SS, somatosensory areas; AU, auditory areas; AA, postparietal association cortex; TH, thalamus. A more detailed description of the brain regions is provided in Supplementary Table 1.

First, the activation patterns while stimulating the right hand were analyzed. Two subjects did not show activation in the primary somatosensory cortex (S1, hand knob), so they were excluded from further analysis regarding Experiment 2. The activation maps of the tactile stimulation of the second-level analysis were defined as ROIs (primary and secondary somatosensory cortex) in the extraction of the time series (S1 and S2 in Supplementary Table 1). A summarized illustration of the GCA is shown in Figure 3. A more detailed illustration of the matrix representation of the pairwise causality results is included in Supplementary Figure 1.The results of the causality analysis revealed two basic patterns of information flow in the investigated regions. For *RN* compared to *WN*, a direct flow of information from the thalamus to the somatosensory cortex is prominent. In acoustic stimulation with *WN*, on the other hand, a complex interaction of all involved network partners becomes apparent. In particular, an intense information exchange between the auditory cortex, the integrative association areas in the parietal cortex, and the thalamus emerge. The somatosensory cortex continues to receive input primarily from the thalamus, but now directly exchanges information with the auditory system and the association cortex. Significance level at *p* < 0.05, FWE-corrected.

## Discussion

The results of our study showed that acoustic stimuli such as sounds generally perceived as *RN* or *WN* change the perception of a touch on the skin. More importantly, both categories of noise have opposite effects on the perception of touch. *RN* improves the perception while *WN* impairs it. Additionally, the brain activity under application of these two types of noise again reveals significant differences. In the case of *WN*, brain activity increases compared to *RN* in brain regions belonging to the auditory, visual, somatosensory, and integrative systems. This is also associated with significantly increased network activity during the processing of a simple touch on the skin. Networks outside the somatosensory system are particularly affected. Communication between the thalamus, integrative brain areas, and the auditory system are amplified. In the presence of *RN*, however, this network activity decreases significantly and is replaced by a more unidirectional flow of information from the thalamus to the somatosensory cortex. The discussion of our findings will mainly focus on two topics: the nature of crossmodal interactions and the effect of noise on sensory perception.

### Processing of Touch: Uni- and Multisensory Processing

Simple, non-painful touch is presumably processed *via* two distinct pathways (Dijkerman and de Haan, 2007). Both begin in the thalamus, projecting to the primary somatosensory cortex (S1) (Brodmann areas 3a, 3b, 1, and 2). From there, they either pass through S2 into the posterior insula or terminate in the posterior parietal cortex (PPC). A good overview is presented by Klingner et al. (2011). These two pathways represent the classical hierarchical processing of somatosensory information. Recognition and perception take place in both target areas (posterior insula and PPC). However, there is an increasing departure from this classical view in which primary sensory cortices process only one specific modality of sensory stimulus processing. Many recent studies show that there is interconnectivity between primary sensory cortices of different modalities. This observation raises the question of whether any cortex can be truly unisensory (Macaluso and Driver, 2005; Ghazanfar and Schroeder, 2006). Accordingly, the processing of one sensory modality would automatically have effects on the processing of other modalities. In one of our earlier works, we were able to show that, even in the absence of a visual stimulus, opening and closing the eyes can switch between a more uni- or multisensory oriented processing pathway (Brodoehl et al., 2013). Another general observation is that weak stimuli are in particular receptive to multisensory interactions (Stein et al., 1993; Macaluso and Driver, 2005; Stein, 2012).

### Interactions of Hearing and Touch

There are many examples in which conflicting sensory information can lead to misinterpretations. Furthermore, there is concrete evidence that hearing certain sounds (e.g., scratching one’s fingernails across a blackboard) (Halpern et al., 1986) or seeing certain scenes (e.g., when a spider walks across a neck) (Keysers et al., 2004) can trigger corresponding activity in the somatosensory system. While numerous studies are describing auditory-visual and visual-tactile interactions (Kennett et al., 2001; Vroomen et al., 2001; Ro et al., 2004) there are relatively few that examine interactions between sound and touch. Although there are reports of interactions (Gescheider et al., 1969; Gillmeister and Eimer, 2007; Navarra et al., 2007; Serino et al., 2007), little is known about the physiological principles. Yet some aspects of hearing and touch, such as vibration, seem particularly close. Loud vibrations of a car can be heard and felt. In addition to the apparent proximity of important anatomical structures (S2 and auditory cortex), neuroimaging studies have shown that there are direct interactions between somatosensory and auditory stimuli in the auditory cortex (Foxe et al., 2000). Like between the visual and auditory systems (Falchier et al., 2002), there are direct anatomical connections between the somatosensory cortex and the auditory cortex (Schroeder et al., 2001).

That sound can alter the perception of somatosensory stimuli has already been compellingly demonstrated by Ro et al. (2009). In their experiments, they used a 500 Hz sound and were able to show that the perception threshold for an electrical stimulus on the middle finger of the left hand is improved when sound and somatosensory stimulation occur simultaneously. Besides, they showed that there was a clear spatial effect. The improvement in recognition performance occurred only when the acoustic stimulus was also presented on the corresponding side of the body.

Major differences from our study should be highlighted: the 500 Hz tone used by Ro et al. (2009) was played in synchrony with the somatosensory stimulus. In our study, the acoustic sounds were played as a sustained stimulation. The 500 Hz tone itself can be classified as very uniform and of high frequency. We used a real touch on the skin in our experiments (both in the MDT and in the fMRI experiments). Typically, electrical stimulation is considered artificial (Burke and Gandevia, 1988; Dean et al., 2006). We want to emphasize these differences because partially contradictory results (to those of Ro et al., 2009) emerged in our study. The results of Ro et al. (2009) could be interpreted as a consequence of a temporal and spatial orientation of alertness, analogous to Spence and Driver (1997) and similar to cueing mechanisms. However, this is not the case for our results, where there is no direct temporal and spatial relationship between auditory and tactile stimuli. However, we were able to show that two different types of noise had different effects on the perception of touch and brain activity.

### General Effects of Noise on Brain Activity

Certain types of noise can have a calming effect on humans. Othman et al. (2020) exemplified that *WN* can lead to an improvement in auditory working memory. This was associated with significantly increased activity in the auditory system, cingulate, and frontal brain, among others. The main argument here was that *WN* can create an ideal configuration of background noise in the brain (Faisal et al., 2008). Positive effects of *WN* have also been shown for other cognitive functions (Soderlund et al., 2010). But there is only very limited evidence for the benefit of the most widespread use of *WN* in our population: supporting sleep initiation (Hong et al., 2021; Riedy et al., 2021). Not surprisingly, studies show negative effects of long-term everyday noise exposure (e.g., traffic noise), especially for the cognitive development of children (Stansfeld et al., 2005; Szalma and Hancock, 2011; Klatte et al., 2013; Schlittmeier et al., 2015). Additionally, there is no doubt about the adverse effects of long-term noise exposure especially on the cardiovascular system (Maschke, 2011; Munzel et al., 2014, 2018).

### How Noise Can Improve Attention and Perception

In a recent study by Schlittmeier et al. (2015), the effects of different noise levels of traffic sounds on attention-based cognition tasks were investigated. Here it was shown that moderate noise (of 50 dB) led to an improvement in a mental arithmetic task, whereas this effect did not occur at 70 dB. At 70 dB there was even a deterioration in the Stroop test, which is a well-established word-color-interference test. The main explanation given here is that traffic noise leads to an increased attentional focus, which has beneficial effects on performance in the arithmetic task and, in contrast, negative effects on performance in the Stroop test (Kahneman, 1973; Broadbent, 1978; Smith and Broadbent, 1981). This increase in phasic attention may have contributed to the improved perceptual performance of touch in the condition with *RN* as described in our present work.

Under normal circumstances, noise is perceived as a disturbance. In this context, an improvement of the signal-to-noise ratio is often considered desirable. However, in certain, often non-linear, systems, noise can help to amplify weak signals. This phenomenon is called stochastic resonance (Wiesenfeld and Moss, 1995). This is particularly relevant for the processing of sensory stimuli since external stimuli are always affected by either thermodynamic or quantum mechanical effects due to their nature (Faisal et al., 2008). It has been shown that the perception of a sensory stimulus can be significantly enhanced by a certain level of noise. Here, however, noise is related to the specific stimulus itself and not an acoustic stimulus. Noise around a subthreshold tactile stimulus can act as a kind of negative marker and increase the chance of perceiving that weak stimulus (Moss et al., 1996). With the auditory system, it has also been shown that certain levels of noise can lead to improved perception and discrimination of acoustic signals (Zeng et al., 2000). These findings were obtained both with purely acoustic stimulation and with direct electrical stimulation.

Noise in one system can enhance perception in a different modality, as has been shown in the context of *WN* and visual perception (Gleiss and Kayser, 2014). This has been investigated for the first time for the interaction of auditory and somatosensory systems in our work.

### Results of This Study in the Context of Crossmodal Interactions, Shifting Attentional Focus, and Stochastic Resonance

The analysis of brain activity in our study indicated that cerebral activity differs between the two acoustic stimuli (*WN* and *RN*). It is, however, a problem that no reliable baseline activity could be defined since no rest condition (without noise) could be realized in the scanner room due to the scanning noises (Shellock et al., 1998). We interpret our results in a way that the unpleasant *RN* creates a brain state with an optimized unimodal procession of somatosensory stimuli. This might be favored by focused phasic attention (Schlittmeier et al., 2015). This results in a lower perception threshold as demonstrated by the MDT. The *WN* environment, on the other hand, led to significantly increased activity and connectivity in the auditory and somatosensory cortex, the association cortex, and the thalamus. At first, it may appear contradictory that this results in a decline in the perceptual performance of touch. However, some of our previous work has shown that increased connectivity of sensory and integrative brain areas, while not associated with the improved perceptual performance of simple stimuli, can lead to improved processing of more complex stimuli, that involve higher hierarchies of sensory and integrative processing (Brodoehl et al., 2016).

### Shortcomings of Our Trial

To address limitations of our trial, the constricted comparability between passive fMRI experiments and active somatosensory perception experiments must be discussed. Active participation of subjects while in the MRI scanner would involve artifacts through movement and presume additional personal in the scanner room during image acquisition. This limitation could be handled by physical tools which present a somatosensory stimulus on one hand while providing the possibility for active feedback on the other hand. Furthermore, *WN* could trigger a more pronounced cerebral activation because it includes the whole frequency range, and the auditory cortex is organized tonotopically. However, this aspect is still a matter of discussion and not yet clarified. Another possibly relevant aspect is the difference in valence and structure of the applied acoustic stimuli. *WN* might offer a calming effect upon some participants, especially in contrast to the chosen *RN*. An additional acoustic stimulus with positive valence and even structure would help distinguish the detected effects and offer control of this aspect.

## Conclusion

The current data provide evidence for a behavioral relevant influence of acoustic noise on the cerebral processing of somatosensory information. Depending on the nature of acoustic noise we found contrary effects with increased perceptual sensitivity due to *RN* and decreased sensitivity due to *WN*. Our further analyses of the cerebral information processing provide evidence that interactions of acoustic and somatosensory stimuli occur at multiple levels in a complex and spatial distributed network. Subsequent studies could investigate changes in information processing while experiencing different acoustic stimuli.

## Data Availability Statement

The raw data supporting the conclusions of this article will be made available by the authors, without undue reservation.

## Ethics Statement

The studies involving human participants were reviewed and approved by the Ethics Committee of the Medical Faculty of the Friedrich Schiller University Jena, Germany. The patients/participants provided their written informed consent to participate in this study.

## Author Contributions

DS and SB: conceptualization. SB, DS, and MN: methodology, investigation, and writing – original draft. MN and SB: visualization. OW, SB, and CK: supervision. MN, DS, SB, CK, and OW: writing – review and editing. All authors contributed to the article and approved the submitted version.

## Conflict of Interest

The authors declare that the research was conducted in the absence of any commercial or financial relationships that could be construed as a potential conflict of interest.

## Publisher’s Note

All claims expressed in this article are solely those of the authors and do not necessarily represent those of their affiliated organizations, or those of the publisher, the editors and the reviewers. Any product that may be evaluated in this article, or claim that may be made by its manufacturer, is not guaranteed or endorsed by the publisher.

## Footnotes

1
http://www.fil.ion.ucl.ac.uk/spm
