# Supplementary material for: Acoustic Stimuli Can Improve and Impair Somatosensory Perception
Source: Front Neurosci. 2022 Jun 23;16:930932. doi: 10.3389/fnins.2022.930932 (PMC9259856; doi:10.3389/fnins.2022.930932)
Supplement: Supplementary file 1 [file Table_1.DOCX]

Supplementary Material

# Supplementary Figures and Tables

## Supplementary Figure S1: Detailed results of the GCA (Granger causality analysis)


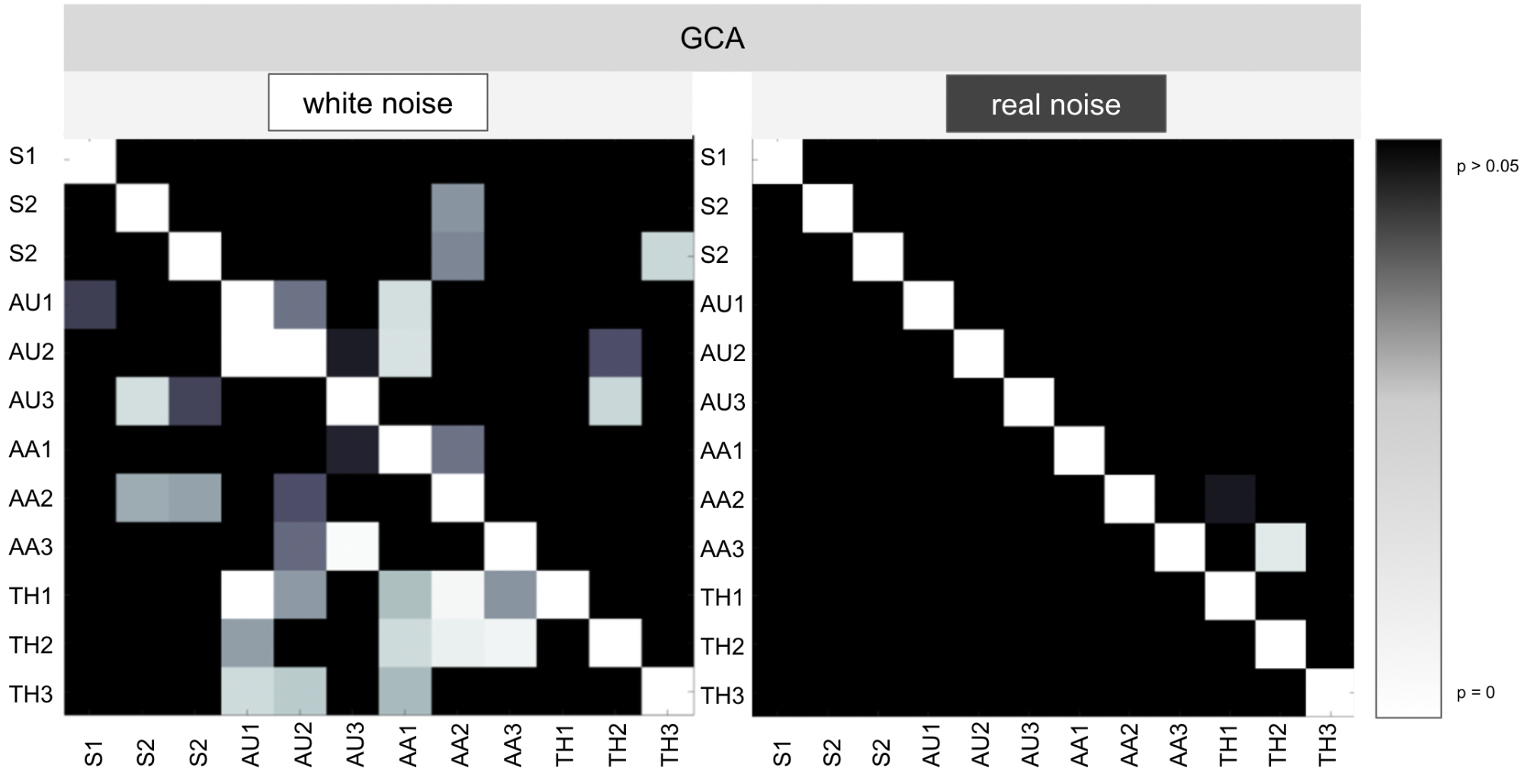


Note: Pairwise results of the group comparison of the Granger causality analysis of fMRI experiment 2. The p-values of the pairwise comparison of "white noise > real noise" (white noise) and "real noise > white noise" (real noise) are shown. T-values were calculated using non-parametric pairwise T-tests. Values with a p-value ≤ 0.05 are highlighted differently from black. Labels: S1/2 - primary and secondary somatosensory cortex, AU1-3 - auditory cortex, AA1-3 - parietal association cortex, TH1-3 - thalamus subregions; a more detailed description of the regions involved in the GCA are provided in Table S1 (supplementary).

## Supplementary Table S1: Regions of interest defined for analysis of effective connectivity

| Label | MNI |  |  | Description | 2D slice |
| --- | --- | --- | --- | --- | --- |
|  | X | Y | Z |  | (coronar) |
| S1 | -51 | -19 | 16 | primary somatosensory cortex | 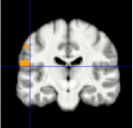 |
| S2/S2 | -36 | -11 | 10 | secondary somatosensory cortex | 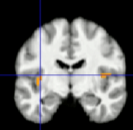 |
| AU1 | -51 | -19 | -8 | gyrus temporalis superior | 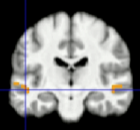 |
| AU2 | -60 | -22 | -11 | gyrus temporalis inferior | 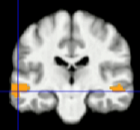 |
| AU3 | -42 | -23 | -23 | gyrus temporalis medius | 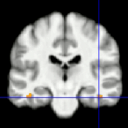 |
| AA1 | -30 | -37 | 58 | superior parietal lobulus (SPL) | 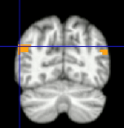 |
| AA2 | -51 | -70 | 28 | inferiorer parietaler lobulus (IPL) | 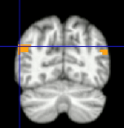 |
| TH1 | -15 | -25 | 1 | thalamus, parietal | 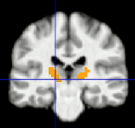 |
| TH2 | -13 | -33 | 4 | thalamus, temporal | 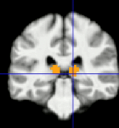 |
| TH3 | -18 | -28 | 7 | thalamus, postparietal | 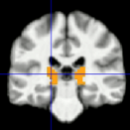 |

Note: Representation of ROIs for granger causality analysis. Each region consists of 500 voxels (100 for the thalamus) symmetrically on the left and right hemispheres.
